# Establishing a Care Continuum for Cardiometabolic Conditions for Patients with Serious Mental Illness

**DOI:** 10.1007/s11886-023-01848-z

**Published:** 2023-02-27

**Authors:** Karly A. Murphy, Gail L. Daumit

**Affiliations:** 1 Division of General Internal Medicine, University of California San Francisco School of Medicine, 1701 Divisidero Street, Suite 500, 94117 San Francisco, CA USA; 2 Division of General Internal Medicine, Johns Hopkins School of Medicine, Baltimore, MD USA

**Keywords:** Serious mental illness, Schizophrenia, Cardiovascular disease, Diabetes, Integrated care, Primary care

## Abstract

**Purpose of Review:**

Addressing cardiometabolic risk factors in persons with serious mental illness requires early screening and proactive medical management in both medical and mental health settings.

**Recent Findings:**

Cardiovascular disease remains the leading cause of death for persons with serious mental illness (SMI), such as schizophrenia or bipolar disorder, much of which is driven by a high prevalence of metabolic syndrome, diabetes, and tobacco use. We summarize barriers and recent approaches to screening and treatment for metabolic cardiovascular risk factors within physical health and specialty mental health settings.

**Summary:**

Incorporating system-based and provider-level support within physical health and psychiatric clinical settings should contribute to improvement for screening, diagnosis, and treatment for cardiometabolic conditions for patients with SMI. Targeted education for clinicians and leveraging multi-disciplinary teams are important first steps to recognize and treat populations with SMI at risk of CVD.

## Introduction

Cardiovascular disease (CVD) is the leading cause of death for persons with serious mental illness (SMI), such as schizophrenia, schizoaffective disorder, or bipolar disorder [1]. It directly contributes to premature mortality by 10–20 years compared with populations without mental illness—unfortunately, this gap has not narrowed in the past few decades [1–3].

Much of this mortality disparity is driven by the high prevalence of cardiometabolic risk factors, tobacco use and low rates of physical activity in populations with SMI [4–7]. Cardiometabolic risk factors (diabetes mellitus, dyslipidemia, hypertension, and overweight/obesity) are a set of risk factors that contribute to the development of CVD [8]. Estimates suggest that people with serious mental illness may have nearly a twofold risk of developing diabetes compared with those without serious mental illness [4]. Estimates of the prevalence of diabetes range from 13 to 18% and 10 to 61% for hypertension in populations with SMI [9, 10]. The use of antipsychotic medication may further elevate their risk of metabolic syndrome through weight gain and altered glucose metabolism [11, 12]. In addition, approximately half of individuals with SMI smoke tobacco, with higher rates observed in individuals with a schizophrenia diagnosis [7, 13]. The goal of this paper is to summarize the known barriers and current strategies to address these barriers in the following domains: screening, diagnosis, treatment, and control of cardiometabolic risk factors. We also include approaches for engaging patients with SMI in disease management who have a diagnosis with a CVD risk factor.

## Continuum of Care for Cardiometabolic Conditions

**Fig. 1:**
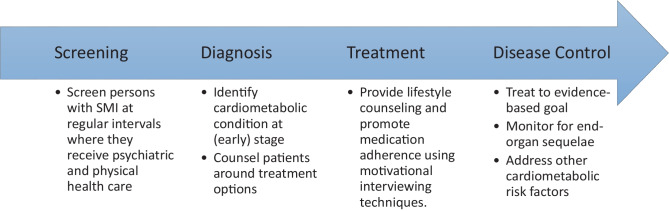
Care continuum for cardiometabolic conditions with the goal of reducing cardiovascular disease mortality for persons with serious mental illness

We focus on cardiometabolic risk factors as all are targeted through early identification and interventions, including overlapping strategies for lifestyle modification [14]. In addition, screening for most of these conditions has long been a recommended priority within psychiatry and general medical care [15]. Management of these cardiometabolic risk factor conditions operates as a continuum of care: screening, diagnosis, treatment, and control (Fig. 1).

### Screening and Diagnosis

The goal of screening is to identify potential diseases, particularly in asymptomatic patients, in order to initiate treatment at an early disease stage and reduce the progression of disease [16]. Evidence shows control of cardiometabolic conditions significantly reduces the risk of mortality [14]. Populations who are at elevated risk of developing disease, such as by demographics (age, race/ethnicity), family history, other comorbid conditions (e.g., chronic kidney disease), or iatrogenic causes (e.g., psychotropic medication), should be screened at earlier and more frequent intervals [14, 17]. Studies indicate that people with schizophrenia are less likely to receive recommended screenings for diabetes, hyperlipidemia, and obesity than those without mental illness [18–21].

### Treatment and Control

Treatment of cardiometabolic conditions focuses on achieving recommended targets for the given risk factor: glycemic control for diabetes, blood pressure control for hypertension, reduction in blood cholesterol for dyslipidemia, and weight reduction for obesity [14]. In many studies, patients with SMI are less likely to receive guideline-concordant care for these cardiometabolic conditions than those without SMI [6, 21]. In one study of 1433 participants with schizophrenia, 70% of individuals with diabetes received hypoglycemic agent, but only 38% of individuals with hypertension received pharmacologic treatment and 12% of individuals with dyslipidemia received pharmacologic treatment, such as with a statin [22]. In another study, individuals with schizophrenia and diabetes were less likely to receive recommended lipid testing, eye exams, monitoring of hemoglobin A1c values compared with individuals without schizophrenia [21]. Yet in other studies, no differences in rates of guideline-concordant care were observed. However, many of these studies noted overall low rates of guideline-concordant care, which suggests that there is need for improvement in quality metrics. Rates also appear lower in Medicaid populations and higher in Veteran’s Administration populations, suggesting additional variation across health insurer and healthcare delivery system [6].

## Barriers to Care

### Patient-Level Barriers

Patients with SMI are likely to experience disadvantages of low socioeconomic status, low educational attainment, unemployment, social isolation, homelessness, criminal justice involvement, and substance use disorder, all of which are associated with an increased risk of developing cardiometabolic conditions [23–26]. They may also be more likely to live in congregate settings, with limited control of menu planning and choice of healthy foods.

Patients with schizophrenia may have cognitive dysfunction or experience disorganized thought processes as part of a psychotic disorder and are among the fastest growing subgroup of beneficiaries of disability [25, 27]. Such cognitive difficulties may only add to patient challenges in navigating a complex healthcare system [28]. Furthermore, patients with SMI, as with those without SMI, require knowledge of how to self-manage their underlying cardiometabolic conditions. They may benefit from additional educational sessions with information broken into small components [29, 30]. Cognitive challenges could influence a patient’s ability to schedule recommended testing and follow-up. As much of chronic disease care relies on regular follow-up (e.g., monitoring of blood pressure, hemoglobin A1c), delays in care may result. Studies report an association between delays in seeking medical care and SMI status [31, 32].

### Provider-Level Barriers

Challenges in who provides physical healthcare and how it is implemented are observed from both primary care and psychiatry perspectives. Primary care physicians and psychiatrists envision a shared responsibility for the care of patients with SMI and want a shared consultative model to implement such care in both primary care and mental health settings [33–36]. However, in many settings, there remains a lack of a clarity as to how such responsibility is divided and the specific roles of each provider [33, 36].

Physical healthcare providers report a lack of time to address screenings and treatment plans and feel less prepared to engage in shared decision-making with patients with SMI who may also experience communication challenges [37–39]. Specifically, some have noted that they feel concerned about their ability to assess a patient’s capacity to make decisions or how to discuss follow-up plans for future health scenarios [40]. Primary care physicians report challenges with communicating if patients with SMI have significant psychiatric symptoms that they feel are not well controlled [40, 41].

Importantly, many patients with SMI may see their psychiatrists or other mental health clinicians more frequently than a primary care physician [42]. Persons with SMI are less likely to be seen in primary care clinics than those without SMI [43, 44]. Yet, many psychiatrists report a lack of a systematic approach for screening for cardiometabolic conditions and insufficient time to incorporate screenings into their practice, particularly when patients have psychiatric illness needs [34, 35]. Others express concern that they lack sufficient training and it is out of their scope of practice. Psychiatrists also report challenges in arranging medical follow-up [35].

Persons with SMI also report experiencing stigma from healthcare providers, which may act as an additional deterrent to engaging with healthcare providers, attending visits, and adhering to recommended treatment plans [39, 41, 45]. The stigma that they may experience may reflect implicit biases around mental illness [46, 47]. Implicit bias is an automatic, unintentional process, where stereotypes are activated and influence judgments and behaviors [46]. In other marginalized populations, implicit bias has been associated with differences in acute pain management, treatment of myocardial infarction, and asthma exacerbations [46, 48]. In a study of 166 healthcare providers, participants who endorsed stigmatizing characteristics of a patient with schizophrenia were less likely to refer to specialist care, refill non-steroidal pain medication, and to endorse that patient would not be adherent to treatment for low back pain [37]. However, few further studies on implicit bias and clinical decision-making have been conducted with an SMI population.

In practice, healthcare providers, whether through implicit biases or being unaware of additional risk factors, may be underestimating the risk of CVD in populations with SMI and therefore under-treat this population [22]. In one US-based health system, the mean 10-year risk of developing ASCVD was significantly higher in populations aged 40–75 years with SMI compared with those without SMI [9]. Moreover, this risk of developing cardiovascular disease is not isolated to older adults. When a 30-year risk of developing CVD was calculated using the Framingham risk score in adults ages 18–59 years, as many as 25% of adults with SMI were observed to be in the highest risk category compared with 11% of adults without SMI [9]. In addition, the use of antipsychotic medication may be an additional risk enhancer for CVD independent of traditional of other cardiometabolic risk factors; it is captured in CVD risk calculation scores in the UK but not in the USA [11, 12, 14, 49].

### System-Level Barriers

System-level and structural barriers disproportionately affect populations with SMI, which likely strongly influence both the prevalence and management of cardiometabolic risk factors [23, 28]. First, the physical and mental healthcare systems have traditionally been siloed apart from one another, contributing to fragmentation of care [50]. Physical healthcare providers and mental healthcare providers may not share electronic health records, much less co-located, physical space-key attributes that promote coordination of care across providers [51]. Second, many persons with SMI face structural barriers, such as lack of transportation to medical appointments and limited financial resources for medication and appointment copayments [28]. Approximately 20–25% of the population who experience homelessness also have a diagnosis of SMI [52]. There also remains limited support for individuals with disabilities through employment opportunities, social programs, and community resources [53].

A majority of people with SMI have insurance, with most being publicly insured [54]. However, younger individuals with schizophrenia may have more frequent and longer gaps in insurance coverage (“churn”) and primary care access than those without schizophrenia [55, 56]. Within 1 year, 54% of individuals aged 18–34 years with schizophrenia were noted to have a disruption in insurance compared with 37% of similarly aged individuals without schizophrenia [55]. As a consequence of gaps in insurance, there may be delays in seeing medical providers or attaining recommended screening tests. In addition, access to mental healthcare remains a barrier, with community mental health clinics being underfunded and only a limited number of psychiatrists available within a given insurance network [57].

## Strategies to Address Barriers

Given the multi-level barriers present to care for individuals with SMI with cardiometabolic conditions, current strategies have focused on how healthcare is organized and delivered, with an emphasis on early identification of cardiometabolic conditions through place-based and population health-based strategies.

### Organization of Healthcare Delivery

#### Bring Physical Health Services to Mental Health Programs

As populations with SMI are frequently seen at mental health clinics, delivery models (e.g., reverse-integration programs, health homes) have sought to bring physical health services to specialty mental health programs [20, 58–61]. Over the past decade, these models have increasingly been implemented in the USA through Substance Abuse and Mental Health Services Administration’s (SAMHSA), a branch of the US Department of Health and Human Services Primary Behavioral Health Care Integration (PBHCI) program and the Affordable Care Act Medicaid health home waiver [20, 60]. The Medicaid health home waiver allows specialty mental health programs to bill for care management and care coordination services, which traditionally has not been reimbursable; to date, 17 US States and the District of Columbia have implemented this waiver program for health homes [62]. Models have been implemented in three ways: (1) co-located primary care providers embedded within mental health clinic or team; (2) psychiatrists initiate management of common chronic conditions using algorithms with a primary care physician acting as consultant, or (3) [nurse] care manager tasked with coordinating physical healthcare but embedded within mental health setting [20, 34, 59, 63, 64].

In the first model, primary care physicians and psychiatrists are co-located onsite within a community mental health center [65]. This model allows primary care physicians and psychiatrists to deliver their usual practice and to collaborate when needed for mutual patients [65, 66]. However, this model is uncommon, with less than a quarter of community mental health sites offering integrated primary care [66]. Facilities likely need a high level of resources to implement and support delivery of primary care (e.g., quality, accreditation, availability of wellness services), which itself may be a high barrier for sites serving smaller number of patients [20, 66].

In the second model, psychiatrists are tasked with screening for common cardiometabolic conditions, counseling patients to reduce risk of cardiometabolic conditions, limiting side effects from psychotropic medications, and to initiate treatment for common cardiometabolic conditions (e.g., metformin for diabetes) until patients can be connected to general medical services [34, 36]. These programs have the advantage to reach populations with SMI who are actively engaged with their mental healthcare providers and reduce the burden of patients needing to go elsewhere to receive their physical healthcare [20, 58, 59, 61]. However, some psychiatrists express discomfort that treatment of cardiometabolic conditions is out of their skillset [35, 67]. More training and support is needed for psychiatry trainees and practicing psychiatrists, including cross-training opportunities for psychiatrists to work collaboratively with general medical and social services [34, 36]. In addition, this model depends on referral for physical health services after screening/diagnosis and does not address long-term treatment and control of cardiometabolic conditions for patients with SMI [34].

In the third model, a care manager, often a nurse, is embedded within a community mental health clinic [63]. The care manager acts to coordinate across physical health (often primary care) and behavioral health services and deliver care management services [68]. Notably, primary care is not co-located in this model and often is in a separate health system. One clinical trial intervention successfully leveraged a nurse care manager and health coaches, who were embedded within community mental health clinics, to reduce overall CVD risk for adults with SMI and at least one CVD risk factor [69••]. Here, the nurse developed an individual care plan, worked with participants around their individual health goal, and coordinated care. The health coaches delivered education around health behaviors related to CVD risk with material edited for improved readability given that many participants experienced cognitive dysfunction and low health literacy [25]. Continual communication, regular check-ins and follow-up, accountability, and support of patient goals were identified as key components [70]. However, in non-clinical trial settings, results have been more limited, particularly those funded through the public insurance-based, Medicaid waiver health home program [20]. Programs report improvement in screening rates of cardiometabolic conditions but no improvement in treatment of cardiometabolic conditions, such as glycemic control or blood pressure control [20]. Programs face barriers around communication when the care manager is not able to see existing electronic health records and must rely on phone calls or faxes to obtain updated medical records [63]. Such barriers may further contribute to miscommunication or delays in care. Some programs also report a lack of capacity for population health management, a staple nowadays in chronic disease care and monitoring [20, 71]. Greater investment in information technology, workforce development, and financing is required [72].

#### Support Physical Healthcare Clinics with Mental Health Professionals

Persons with SMI also report wanting to be seen in primary care [41]. The Collaborative Care Model (CCM) incorporates a psychiatrist and other mental health professionals into a primary care practice, with early studies focusing on providing mental health treatment [73–75]. However, later studies have examined whether CCM could improve delivery of physical healthcare for patients with SMI [76, 77]. In one small study, patients with psychosis and poorly controlled diabetes were assigned care to a team featuring a nurse care manager, psychiatrist, primary care provider, and endocrinology consultant. Diabetes education was delivered using motivational interviewing and behavioral activation strategies and material was adapted to address specific needs of patients with psychosis. After 3 months, mean hemoglobin A1c declined significantly for intervention-arm participants (9.4% to 8.0%) but did not significantly change for control arm participants (8.3 to 8.0%) [77]. No differences were observed for blood pressure control. In this model, participants also received mental healthcare at the CCM.

Facilitators of this model include the presence of a team member with a specialist mental health background (e.g., care manager, care provider) who communicates effectively with primary care providers and receives support from healthcare clinic/system and patients with SMI who are open to working with the care manager [78]. Care providers could enhance a primary care physician’s understanding of a patient with SMI’s concerns, while a primary care physician could increase the care manager’s understanding of why a specific chronic condition should be prioritized. In turn, the care provider could then help to introduce these topics to the patient. Mean healthcare costs are higher for patients with SMI compared with SMI, particularly if they have a common chronic condition, such as diabetes or chronic kidney disease [79]. Given that primary care practices may have a small proportion of patients with SMI and limited resources, practices may wish to direct CCM resources towards patients with SMI who also have a major chronic condition.

Barriers occur when care managers lack the support of supervisors or primary care physicians, lack the knowledge or skills required to deliver chronic disease education, or struggle with use of information technology, which is often a primary means of communication [78]. More work is needed to understand the training, education, and infrastructure needed to support healthcare professionals who may not be used to working with patients with SMI.

### Strategies to Support Healthcare Providers

#### Use of Clinical Decision Support Tools

Clinical decision support systems leverage informatics systems (e.g., electronic health records) to assist clinicians with delivering evidence-based care by taking existing information on a specific patient to recommend specific screening tests, medications, and treatment goals [80]. In a recent trial of adult patients with SMI (defined as schizophrenia, schizoaffective disorder, or bipolar disorder), 76 primary care clinics were randomized across three health systems for primary care physicians and psychiatrists to receive best practice advisory prompts based on a patient’s modifiable CVD risk profile [81••]. Across the 12-month intervention, the intervention was associated with a net 4% decrease in total modifiable CVD risk. Investigators calculated this intervention could prevent 3 events per 1000 patients. Patients who were younger (aged 18–29 years) or middle-aged (aged 50–59 years), Black or White were more likely to benefit from the intervention; however, no significant treatment effects were observed for individual CVD risk factors [81••].

One benefit of these decision support systems is that they can be implemented within any specialty (e.g., internal medicine, psychiatry), modified according to relevant populations, and used in combination with other interventions, such as team-based care models [80]. Best practice alerts in other settings have been shown to improve laboratory-based metabolic monitoring while a patient is prescribed antipsychotic medication [82, 83]. However, studies note that clinicians may override alerts due to fatigue from overuse of alerts or having alerts that are not well targeted to specific patients [84]. Future work is needed to understand how clinical decision supports may be used with populations with SMI, who may be at risk of under-diagnosis of ASCVD [9].

#### Leverage Multi-disciplinary Teams

As described earlier, use of multi-disciplinary teams where the healthcare is delivered (primary care clinic vs mental health clinic location) is increasingly being leveraged to care for populations with SMI around CVD risk factors. Programs using individual and group weight management sessions have been shown to be effective in populations with SMI [85]. Yet work is needed around how to adapt existing evidence-based programs (e.g., diabetes prevention program, weight management) in real-world settings [86].

Employing educators who are not physicians to deliver critical health education also may address known time shortages within traditional clinic visits. One common theme is to leverage healthcare team members who have experience working with persons with SMI and who can help to educate other healthcare team members around specific care needs that populations with SMI may require [59, 63, 77, 78]. Health coaches can help individuals with SMI learn how to identify their health goals and to self-manage cardiometabolic conditions [69••]. One ongoing intervention is training direct care staff at specialty mental health clinics to conduct evidence-based CVD care coordination through a team-based implementation strategy [87]. All may use motivational interviewing to deliver this educational material [88].

Another resource are peers with lived experience with SMI. In one study, they have been shown to promote self-management and self-efficacy around diabetes management [89]. In another study of 400 patients across 6 months, peers helped to positively engage participants around self-management of chronic conditions and improved quality of life [90]. Peers may be powerful role models for patients through relating to their challenges and offering advice and promoting engagement in self-management programs [91]. Self-management programs around diabetes are feasible and acceptable to patients with SMI; however, little is known about their effectiveness as many trials exclude participants with SMI or have limited data available [92, 93].

#### Addressing Unconscious Bias

While stigma and implicit bias are known barriers, there is limited data as to how to address stigma and implicit bias among medical professionals [39, 41]. Contact-based interventions between groups with and without lived experience with SMI has been one strategy; in these interventions, a meeting (“contact”) is set up between an individual from the stigmatized group (here, individual with SMI) and individual from general population (here, medical professional) [94]. It often includes an educational component and occurs in an artificial setting [94]. However, available studies may lack the rigor of a control arm, are of limited duration, or have not been linked to behavioral changes [94, 95]. Studies have used anti-stigma educational training programs, increased contact with individuals with SMI (in-person, video), technology (e.g., virtual reality), and public activism approaches [95–99]. More rigorous studies targeting behavioral change are needed to elucidate mechanisms that both mediate stigma and promote change around caring for persons with SMI. In addition, more education is needed, including in medical school and residency for all specialities given that patients with SMI are at elevated risk for experiencing an adverse events during a medical-surgical hospitalization [100]. However, given the outsize proportion of premature mortality attributed to cardiometabolic conditions, such training is critical for psychiatry and general medical trainees who are more likely to care for this population.

## Future Directions

While CVD remains a leading cause of mortality for persons with SMI, delivery models are increasingly focused on how to systematically screen populations and connect them to treatment for cardiometabolic conditions. Caring for populations with SMI at high risk for developing CVD is challenging with barriers at the system, provider, and patient level. Consequently, multi-level strategies will be required to address how healthcare is delivered to populations with SMI and how to engage them in evidence-based, patient-centered self-management of these chronic conditions.
